# T-614 Promotes Osteoblastic Cell Differentiation by Increasing Dlx5 Expression and Regulating the Activation of p38 and NF-*κ*B

**DOI:** 10.1155/2018/4901591

**Published:** 2018-02-19

**Authors:** Jinglue Song, Hongli Liu, Qi Zhu, Yutong Miao, Feiyan Wang, Fan Yang, Wenjing Cheng, Yebin Xi, Xiaoyin Niu, Dongyi He, Guangjie Chen

**Affiliations:** ^1^Department of Immunology and Microbiology, Shanghai Jiao Tong University School of Medicine, Shanghai Institute of Immunology, 280 South Chongqing Road, Shanghai 200025, China; ^2^Guanghua Rheumatology Hospital, 540 Xinhua Road, Shanghai 200052, China; ^3^The Fifth People's Hospital of Yuhang District, Hangzhou 311100, China

## Abstract

Rheumatoid arthritis (RA) is an autoimmune inflammatory disease characterized by bone loss. Degree of inflammation has been identified as an important initiator of skeletal damage in RA. Iguratimod (T-614) is an anti-inflammatory agent which has been reported to show the inhibitory effect of bone destruction in RA. However, the role of T-614 in osteoblast differentiation is still not clear. In this study, we intended to find the effect of T-614 on the osteogenesis process. We detected osteogenesis markers and transcription factors associated with osteoblastic lineage and bone formation in the culture of mesenchymal stem cells which differentiate osteoblast. The contents and activity of alkaline phosphatase, levels of collagen type I and bone gla protein, and calcium nodule formation were increased significantly after T-614 treated. Meanwhile, the mRNAs expressions of* Osterix* and* Dlx5* were also found to be increased significantly by real-time PCR. The changes of levels of phosphorylation of p38 and NF-*κ*B were also detected by Western blot. The results showed that T-614 promotes osteoblastic differentiation by increasing the expression of* Osterix* and* Dlx5* and increasing the activation of P38. T-614 could advance the ectopic expression of NF-*κ*B to suppress inflammation, which indirectly inhibits the damage of the osteoblasts.

## 1. Introduction

Rheumatoid arthritis (RA) is a systemic autoimmune disease characterized by chronic erosive arthritis. Inflammation in the synovium of joint and progressive damage of the bone and cartilage are the main pathological characteristics. Patients with RA mainly show joint pain, swelling, stiffness, or permanent destruction, and some of this severe damage can lead to disability and malformation or even systemic manifestation [1].

In the progress of RA, bone damage is an important cause of the patients' dysfunction [2]. Thus, bone rebuilding is crucial in the recovery process. While in bone rebuilding, three pathways, including BMP2-Smads signal pathway, P38-MAPK pathway, and TNF-*α*/NF-*κ*B pathway, play a vital role in osteoblast proliferation and differentiation and the injury-repairing process of bone and cartilage.

Iguratimod (T-614) is a new small molecule compound with anti-inflammation and immune regulation functions which has a definite disease modifying effect in an animal RA model. Some researchers report that T-614's effect relies on suppressing several inflammatory factors and immunoglobulin [3–5]. T-614 can accelerate bone formation by inhibiting osteoclast activation and promoting osteoblast differentiation [6, 7]. However, the mechanism of T-614's effect on promoting osteoblast differentiation is still unclear. Studies show that T-614 increases the expression of Osterix (Osx), which is a zinc-finger transcription factor that was crucial in osteoblast differentiation. Meanwhile, two signal pathways may mediate this process, the recombinant human bone morphogenetic protein-2- (BMP2-) Smads signal pathway and the P38-MAPK pathway. BMP2 induces the expression of Osx, while there are several transcription factors upstream of Osx, such as Dlx-5 and Runx2. The MAPK pathway, mediated by P38, interacts with the BMP2-Smads pathway to improve Osx phosphorylation. In addition, TNF-*α* activates the NF-*κ*B pathway to mediate inflammation. TNF/NF-*κ*B pathway participates in osteoblast proliferation, the induction of osteoblast apoptosis, the inhibition of osteoblast differentiation, and the suppression of matrix proteins.

In this study, we investigated the effect of T-614 on the osteogenesis process. We first tested the content of some osteogenesis markers, such as type I collagen (Col I), bone gla protein (BGP), the contents and activity of alkaline phosphatase (ALP), and calcium nodule formation in the mesenchymal stem cells to help to verify its effectiveness and function in promoting osteoblastic differentiation. Then, we aimed to assess T-614's effect on Dlx5 and P38, which are involved in two pathways of osteoblast differentiation. We also intended to investigate whether T-614 inhibits the TNF-*α*/NF-*κ*B pathway.

## 2. Materials and Methods

### 2.1. Cell Culture

Mesenchymal stem cells (MSC) were isolated from murine bone marrow according to [8]. The cells were cultured in *α*-minimum essential medium (*α*-MEM; Invitrogen) supplemented with 10% fetal bovine serum (FBS; Equitech-Bio, Kerrville, TX) and bFGF (PeproTech) at 37°C in a humidified 5% CO2 incubator for several cycles. For each assay, MSCs were plated at 1 × 10^4^ cells/cm^2^, and various concentrations of T-614 were added with or without rhBMP-2 (Genetics Institute, Cambridge, MA).

### 2.2. ALP Content and Activity Detection

MSCs were treated with rhBMP-2 and/or T-614 for 3 days. For ALP staining, the cells were fixed for 15 min with 3.7% formaldehyde at room temperature after washing with phosphate-buffered saline (PBS). Then, the cells were tested for ALP content by using an ALP staining kit (Nanjing Jiancheng Science and Technology Ltd., China) according to the manufacturer's instruction.

To measure the ALP activity, the cells were washed twice with PBS and lysed in M-PER Mammalian Protein Extraction Reagent (Pierce, Rockford, IL) following the manufacturer's protocol. ALP activity was assayed using p-nitrophenylphosphate as a substrate by the Alkaline Phosphatase Test (Beyotime Biotechnology, China), and the protein content was measured using a bicinchoninic acid (BCA) protein assay kit (Pierce). ALP activity was calculated as the total activity units/the total protein concentration.

### 2.3. Alizarin Red Staining

MSCs were cultured in *α*-MEM supplemented with 10% FBS and 10 mM b-glycerophosphate (Sigma) in the absence or presence of 50 ng/ml rhBMP-2 and 10 *μ*g/ml T-614 for 14 days. The medium was replaced every 3 days. For the deposited calcium staining to detect mineralized nodules, the cells were washed with deionized water after being fixed for 15 min with 3.7% formaldehyde at room temperature and were stained with Alizarin Red S (Sigma, St. Louis, MO) at pH 4.3.

### 2.4. Real-Time PCR

MSCs were cultured in *α*-MEM supplemented with 10% FBS in the absence or presence of 50 ng/ml rhBMP-2 and 10 *μ*g/ml T-614 for 14 days. Total RNA was extracted from the MSCs culture using TRIzol reagent (Invitrogen). First strand cDNA was subsequently synthesized using the Prime Script RT Master Mix kit (Takara). Primer Express software (Applied Biosystems, Foster City) was used to design primers from the published cDNA sequence. BLAST searches were conducted on the primer nucleotide sequences to ensure gene specificity. The levels of mRNA for mouse* ALP*,* Col1*,* BGP*,* Osterix*,* Dlx5*, and *β*-*actin* (house-keeping gene) were detected by quantitative PCR using an ABI HT7900 Sequence Detection System with SYBR Green Master Mix (Applied Biosystems). The thermocycler conditions were composed of an initial holding at 50°C for 2 min and a subsequent holding at 95°C for 10 min, which was followed by a 2-step PCR program at 95°C for 15 s and 60°C for 60 s for 40 cycles. All of the procedures followed the manufactures' protocols, and the data were quantitatively analyzed on a Primer Express software (ABI) and Prime 7900HT sequence detection software. The primers used in the study are listed as follows:*β-actin*, Forward 5′-TTCAACACCCCAGCCATGT-3′ and Reverse 5′-GTGGTACGACCAGAGGCATACA-3′;* ALP*, Forward 5′-CATGGCTTTGGGCAGAAGGA-3′ and Reverse 5′-CTAGCCCCAAAAAGAGTTGCAA-3′;* Col1*, Forward 5′-CAGCCGCTTCACCTACAGC-3′ and Reverse 5′-TTTTGTATTCAATCACTGTCTTGCC-3′;* BGP*, Forward 5′-CTGACCTCACAGATCCCAAGC-3′ and Reverse 5′-TGGTCTGATAGCTCGTCACAAG-3′;* Osterix*, Forward 5′-CGC ATC TGA AAG CCC ACT TG-3′ and Reverse 5′-CAG CTC GTC AGA GCG AGT GAA-3′;* Dlx5*, Forward 5′-TCTCTAGGACTGACGCAAACA-3′ and Reverse 5′-GTTACACGCCATAGGGTCGC-3′. Relative quantification of gene expression was performed using the ABI Prism 7900 Sequence Detection System. SYBR Green Master Mix (ABI, USA) was used for real-time RT-PCR to detect the abundance of PCR products among samples. The *β*-actin gene was used as an endogenous control to normalize for differences in the amount of total RNA in each sample. All quantities were expressed as *n*-fold relative to a calibrator.

### 2.5. Western Blot

To investigate the effect of T-614 on P38 MAPK pathway, the cells were cultured in *α*-MEM supplemented with 10% FBS and 50 ng/ml rhBMP-2 in the absence or presence of 10 *μ*g/ml T-614. The cells were harvested at 1.5, 2.5, 3, 4, and 6 h for detecting the expressions of phospho-p38. Meanwhile, the cells were cultured in *α*-MEM supplemented with 10% FBS and 20 ng/ml TNF-*α* in the absence or presence of 10 *μ*g/ml T-614. The cells were harvested at 0.75, 1, 1.5, 2, and 3 h for detecting the expressions of NF-*κ*B and p-NF-*κ*B. At the end of each time point, the cells were lysed using a radioimmunoprecipitation assay lysis buffer, and a bicinchoninic acid protein assay kit was used to determine the protein concentrations. The proteins were separated by 10% SDS-PAGE and were then transferred onto the polyvinylidene difluoride (PVDF) membranes. The membranes were incubated with 3% BSA in TBST (50 mM of Tris, 0.15 M of NaCl, 0.1% Tween 20, PH 7.6) for 1 h to block nonspecific binging sites and were then incubated with the p38, phospho-p38, NF-*κ*B, and phospho-NF-*κ*B antibodies (Cell Signaling Technology, Beverly, MA, USA) at 4°C overnight, followed by incubation with the appropriate conjugated secondary antibodies. The proteins of interest were visualized using an Immobilon Western Chemiluminescent HRP Substrate and a Tanon-5500 Chemiluminescent Imaging System.

### 2.6. Statistical Analyses

Differences between the groups were determined using Student's *t*-test. A one-way analysis of variance (ANOVA) test was used to evaluate whether a gross statistically significant change existed. All of the results are expressed as the mean + SEM. The *P* values < 0.05 or 0.01 were all considered statistically significant.

## 3. Results

### 3.1. Effects of T-614 on ALP Staining and Activity in MSCs

**Figure 1:**
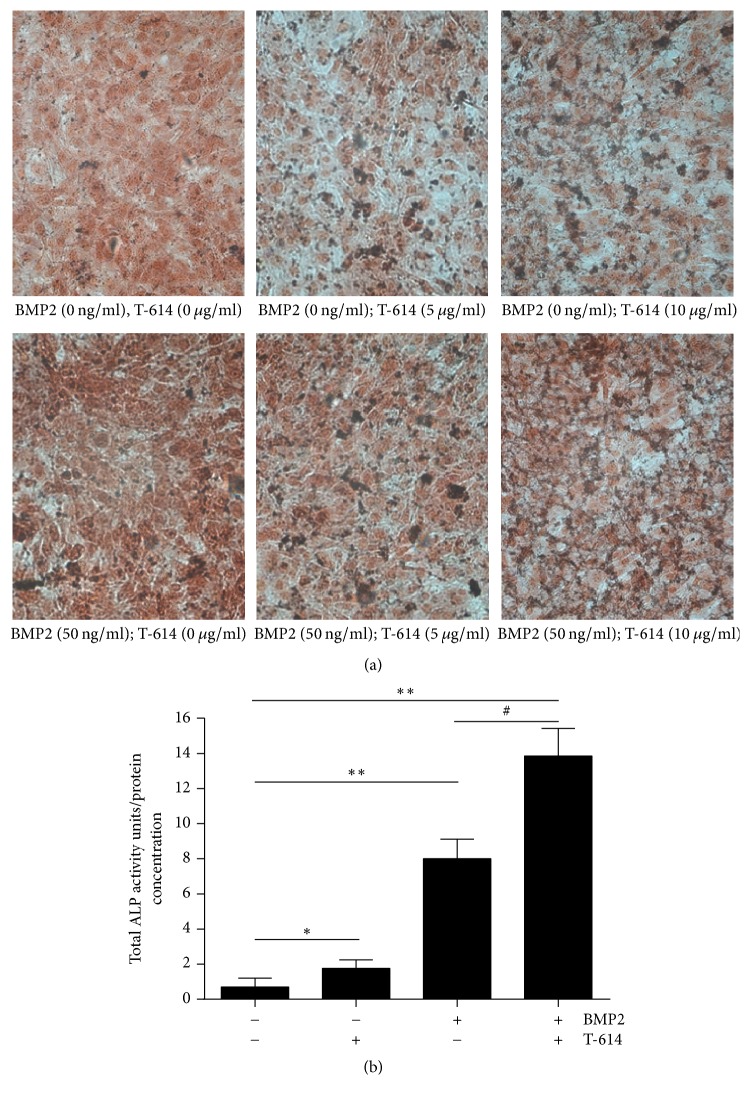
ALP staining and ALP activity in MSCs. MSCs were treated with rhBMP-2 and/or T-614 for 3 days. The cells were tested for ALP content by using an ALP staining kit; ALP activity was calculated as the total activity units/total protein concentration. (a) The stainings are representative of 3 separate experiments with similar results. (b) Statistical analysis of ALP activity. The data are expressed as the means +/− SD (*n* = 3). ^*∗*^*P* < 0.05, ^*∗∗*^*P* < 0.01, compared with the vehicle control; ^#^*P* < 0.05, compared with the BMP2 group.

MSCs are multipotent stromal cells that can differentiate into a variety of cell types [8], including osteoblasts (bone cells), chondrocytes (cartilage cells), myocytes (muscle cells), and adipocytes (fat cells) [9–11]. We cultured MSCs in *α*-MEM supplemented with 10% FBS and bFGF. Alkaline phosphatase, an early phase marker of osteoblastic differentiation, was assayed in the MSCs cultured for 3 days in the presence or absence of rhBMP-2 or T-614. T-614 and rhBMP-2 synergistically increased ALP staining in the MSCs in a dose-dependent fashion (Figure 1(a)). Meanwhile, the increased activity of ALP (total activity units/total protein concentration) in the MSCs is shown in Figure 1(b).

### 3.2. Effects of T-614 on Calcium Nodules Formation in MSCs

**Figure 2:**
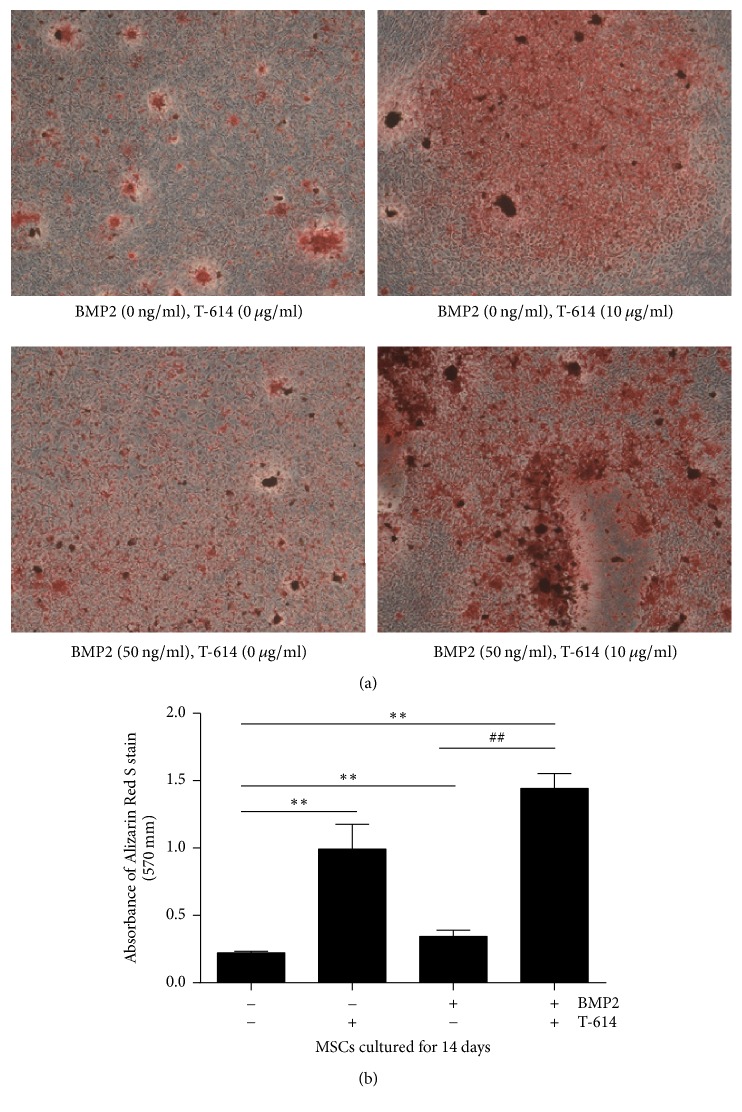
Calcium nodule formation in MSCs. MSCs were cultured in *α*-MEM supplemented with 10% FBS and 10 mM b-glycerophosphate in the absence or presence of 50 ng/ml rhBMP-2 and 10 *μ*g/ml T-614 for 14 days. Then, the calcium content of the mineralized nodules in the cells was stained with Alizarin Red S at pH 4.3. (a) The data are representative of 3 separate experiments with similar results. (b) Statistical analysis of absorbance of Alizarin Red S staining. The data are expressed as the means +/− SD (*n* = 3). ^*∗∗*^*P* < 0.01, compared with the vehicle control; ^##^*P* < 0.01, compared with the BMP2 group.

As shown in Figure 2, in the of absence of 50 ng/m rhBMP-2, less calcium nodules were detected in the MSCs but were increased when compared to the cells cultured with T-614. In the presence of both rhBMP-2 and T-614, more calcium nodules were detected compared with the other conditions.

### 3.3. Effects of T-614 on Osteogenesis Markers and Related Transcription Factors in MSCs

**Figure 3:**
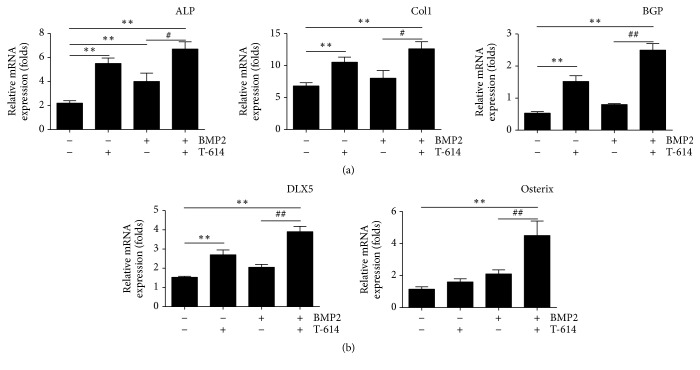
Osteogenesis markers and the related transcription factor expressions in MSCs. The mRNA levels of the osteogenesis markers* Alp*,* Col1*, and* Bgp* in MSCs cultured in the indicated condition (a) and the mRNA levels of the related transcription factors (*Osterix* and* Dlx5*) (*n* = 3) (b) were measured by real-time PCR. The *β*-actin gene was used as a reference to normalize the differences in total RNA in each sample (*n* = 3). Statistical analysis is expressed as the means +/− SD. The data are representative of five experiments with similar results. ^*∗∗*^*P* < 0.01, compared with the vehicle control; ^#^*P* < 0.05, ^##^*P* < 0.01, compared with the BMP2 group.

In the absence of rhBMP-2 and T-614, the mRNA expression of the osteogenesis markers* ALP*,* Col1*,* BGP* of MSCs was less. When the MSCs were treated with T-614, these osteogenesis markers increased significantly. Then, in the presence of rhBMP-2, the mRNA expression of the osteogenesis markers* ALP* and* BGP* of the MSCs cultured with T-614 increased significantly compared to the cells cultured without T-614 (Figure 3(a)), but the expression of Col1 was not changed.

At the same time, we also compared the changes in the mRNA expression of the transcription factors* Osterix* and* Dlx5*. The results showed that, in the absence of rhBMP-2 and T-614, the mRNA expression of* Osterix* and* Dlx5* was less. When the MSCs were treated with T-614, the expression of* Osterix* and* Dlx5* increased significantly. Then, in the presence of rhBMP-2, the mRNA expression of* Osterix* and* Dlx5*, when cultured with T-614, increased significantly compared to the cells that were cultured without T-614 (Figure 3(b)). These results, together with the results of ALP content, activity detection, and calcium nodules formation, indicate that T-614 motivates the osteogenic process.

### 3.4. Effects of T-614 on the p38 and NF-*κ*B Signaling Pathways in Osteoblast Differentiation in MSCs

**Figure 4:**
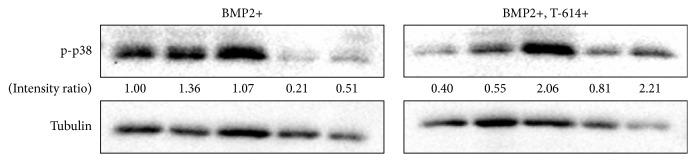
p38 signaling pathway in osteoblast differentiation in MSCs. MSCs were cultured in *α*-MEM supplemented with 10% FBS and 50 ng/ml rhBMP-2 in the absence or presence of 10 *μ*g/ml T-614. Then, the cells were harvested at 1.5, 2.5, 3, 4, and 6 h to detect the expressions of phospho-p38 by Western blot. The data are representative of three experiments with similar results.

After treating the MSCs with rhBMP-2 and T-614 for 1.5, 2.5, 3, 4, and 6 h, the cells were harvested to detect the expressions of phospho-p38. The results showed that T-614 exposure improves the activation of p-p38. At 3 h, the increased level of p-p38 is the highest. The intensity ratio was 2.06 in the presence of rhBMP-2 and T-614, compared to the cells cultured without T-614 where the intensity ratio was 1.07 (Figure 4).

**Figure 5:**
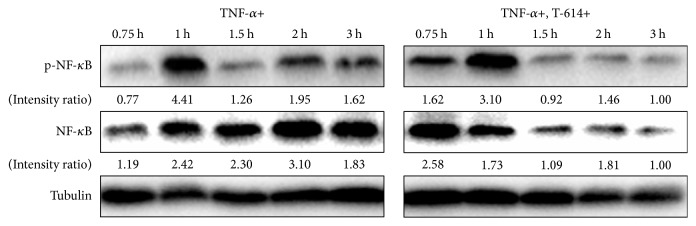
The effect of T-614 on the NF-*κ*B signal pathway. MSCs were cultured in *α*-MEM supplemented with 10% FBS and 20 ng/ml TNF-*α* in the absence or presence of 10 *μ*g/ml T-614. Then, the cells were harvested at 0.75, 1, 1.5, 2, and 3 h to detect the expressions of NF-*κ*B and p-NF-*κ*B by Western blot. The data are representative of three experiments with similar results.

After the MSCs were treated with TNF-*α* and T-614 for 0.75, 1, 1.5, 2, and 3 h, the cells were harvested to detect the expressions of NF-*κ*B and p-NF-*κ*B. The results showed that 1 h of T-614 exposure reduced the expression of p-NF-*κ*b, and T-614 reduced the activation time for NF-*κ*B to reach its maximum.  Figure 5 showed that at 1 h, the level of p-NF-*κ*B reached its peak and persisted for 3 h at the intensity ratio of 1.62 when the MSCs were treated with TNF-*α* only. In contrast, in the presence of TNF-*α* and T-614, although the level of p-NF-*κ*B reached its peak at 1 h, the intensity ratio was lower and decreased quicker.

## 4. Discussion

Iguratimod, also called T-614, is a newly synthesized, multitarget drug that has shown a promising therapeutic effect on rheumatoid arthritis in clinical trials of different time spans (24 and 52 weeks) [12, 13]. It has a clinical efficacy comparable to methotrexate [14] and obtains better clinical results when used, when it is combined in the treatment of cases in which the clinical condition cannot be controlled by methotrexate alone [15].

Studies show that osteoblastic functions are seriously compromised in rheumatoid arthritis patients. At sites of inflammation, the capacity of mineralization of the extracellular matrix is abated, while Runx2, a molecular sign expressed in early differentiated osteoblasts, is found on most cells, and alkaline phosphatase, a molecular sign expressed in middle to late differentiated osteoblasts, is not as abundant. Taken together, bone tissue loss in rheumatoid arthritis cannot be recovered in time [16]. Recently, studies using T-614 have mainly concentrated on its role in immune regulation; however, reports about its effect on osteoclasts and osteoblasts are limited. Bone mass is always maintained through a repeated cycle of destruction and rebuilding. This process is regulated by both osteoclasts, which resorb old bones, and osteoblasts, which form new bones. Our study focuses on the effect of T-614 on osteogenesis. First, we found that T-614 increases the content and activity of alkaline phosphatase and calcium nodules in MSCs. We also found that the mRNA expression of the osteogenesis markers* Alp* and* Bgp* in MSCs cultured with T-614 increased significantly compared to the cells cultured without T-614. ALP, BGP, and Col I are considered markers of mature osteoblasts. However, the expression of* Col1* did not increase as much as* Alp* and* Bgp*. All of these results indicate the T-614 has an effect on osteoblastic differentiation.

Osterix, a transcription factor unique to the mesenchymal lineage, induces the expression of markers in BMSCs associated with the osteoblastic lineage, including bone sialoprotein, proteoglycan, alkaline phosphatase, osteocalcin, and osteopontin [17], and increases alkaline phosphatase activity and the cells ability to proliferate and form calcium nodules [18]. In the study by Kuriyama et al. [7], they speculated that a possible mechanism of the anabolic effect of T-614 might be exerted via the stimulation of Osterix expression, and levels of Osterix expression might account for its different effect on different cell lines. Since the cell line used in our study is a pluripotent stromal cell lineage and the fact that rhBMP can affect the level of expression of Runx2 and Osterix in MSCs, which makes it a potent inducer of osteoblast differentiation [19], we adopted a method that involved adding both rhBMP-2 and T-614 into the BMSC culture. Osterix is upregulated by signaling pathways, such as BMP2 (Runx2, Dlx5), p38-MAPK, Igf1, and ER-stress (lRE1a/XBP1) [20].

Dlx5, a critical transcription factor involved in bone formation, binds to a homeodomain sequence located in the proximal region of the Osterix promoter and, when phosphorylated, Dlx5 recruits p300 to the Osterix promoter to exert its histone acetyltransferase activity, leading to unfolded chromatin and further recruitment of other DNA binding transcription factors or coactivators such as RNA polymerase to start the transcription of Osterix [21]. We found that the mRNA levels of* Dlx5* and* Osx* exhibit the highest expression in the condition that included both T-614 and rhBMP-2. The results indicated that, via the upregulation of Dlx5, T-614 elicits the transcriptional activation of Osterix and further promotes osteoblast differentiation.

P38 MAPK is a member of the mitogen-activated protein kinase superfamily, which is involved in the early stages of osteoblast lineage proliferation via the phosphorylation of Dlx5, Runx2, and Osterix [21]. Our data showed that, although the level of p-p38 in the experimental group (rhBMP-2 with T-614) was relatively lower in the first 3 hours, it reached its peak at 6 h. It consequently prolonged its expression and postponed the climax of p-p38, indicating that T-614 increases the activation of p-38 and, therefore, promotes osteoblastic differentiation.

TNF-*α* is a proinflammatory cytokine that plays a fundamental role in the pathogenesis of rheumatoid arthritis. The actions of TNF-*α* are perceived to be important and include its ability to induce endothelial cell activation, cytokine amplification, osteoclast activation, and osteoblast function impairment, which leads to sustained inflammation and the destruction of bone and the underlying cartilage [22]. As the main downstream molecule of TNF-*α* signal transduction, NF-*κ*B mediates the destruction of osteocytes through the downregulation of the expression and phosphorylation of BMP-Smad1 and the depression of the Runx2 signaling pathway [23]. Via an interaction with the above pathway, NF-*κ*B compromises bone turnover and aggravates clinical conditions. Our data showed that despite the fact that the levels of total and phosphorylated NF-*κ*B were higher in T-614 group in the first 0.75 h after stimulation with TNF-*α*, these remained lower in the next 1 to 3 hours. Thus, by forwarding and shortening the NF-*κ*B activation climax, T-614 keeps the NF-*κ*B activation to a low level in the long run. Yamaguchi et al. reported that a reduction of NF-*κ*B activation alleviates the suppressive action of TNF-*α* on osteoblasts and promotes calcium nodule formation* in vitro* [24]. We can infer that T-614 acts in a similar way.

By estimating the total and phosphorylated NF-*κ*B content through its gray scale, we discovered that the percentage of phosphorylation remains basically the same between the T-614-treated group and the control group, suggesting that T-614 does not influence the phosphorylation of NF-*κ*B substantially. As is reported, during signal transduction, IkappaB kinases phosphorylate the NF-*κ*B p65 subunit on serine 536 in the transactivation domain, leading to a conformational switch, marking the activated status of the p65 subunit [25, 26]. This is also involved in the final ubiquitination and proteasomal elimination of NF-*κ*B [27]. Therefore, we speculate that the possible inhibitory effect of T-614 on NF-*κ*B may involve the suppression of basal NF-*κ*B levels without interfering with NF-*κ*B's separation with I-*κ*b or the phosphorylation process after the separation. This is in accordance with what Aikawa et al. observed in Th-1 cells [28]. As for the fluctuations of total NF-*κ*B in such a short time observed in our study, we speculate that this may be due to nonclassical activation pathways of NF-*κ*B, such as proteasomal elimination after ubiquitination.

Collectively, our data suggest that through the upregulation of Dlx5 and the phosphorylation of p38, T-614 increases Osterix expression, further promotes the differentiation and proliferation of osteoblast precursor cells, and exerts a protective effect under an inflammatory situation by inhibiting the phosphorylated NF-*κ*B level.
